# Interleukin-15 and irisin serum concentrations are not related to cardiometabolic risk factors in patients with type 2 diabetes from Korea and Germany

**DOI:** 10.1007/s00592-019-01417-3

**Published:** 2019-09-10

**Authors:** Kyung Mook Choi, Soon Young Hwang, Kyungdo Han, Hye Soo Chung, Nam Hoon Kim, Hye Jin Yoo, Ji-A. Seo, Sin Gon Kim, Nan Hee Kim, Sei Hyun Baik, Thomas Ebert, Mathias Fasshauer, Matthias Blüher

**Affiliations:** 1 Division of Endocrinology and Metabolism, Department of Internal Medicine, College of Medicine, Korea University, Seoul, Korea; 2 Department of Biostatistics, College of Medicine, Korea University, Seoul, Korea; 3 Department of Biostatistics, College of Medicine, The Catholic University of Korea, Seoul, Korea; 4 Department of Medicine, University of Leipzig, Leipzig, Germany

## Introduction

Physical exercise plays an important role in both the prevention and treatment of cardiometabolic diseases. Peptides secreted or released from skeletal muscle, so-called myokines, contribute to the beneficial anti-inflammatory and insulin-sensitizing effects of increased muscle activity and may, therefore, counteract pathomechanisms of obesity and type 2 diabetes (T2D) [1]. However, the impact of myokines including interleukin (IL)-6, IL-8, IL-13, IL-15, angiopoietin-like 4 (ANGPTL4), fibroblast growth factors (FGF)-2, FGF-21, and irisin on altered organ cross-talk in cardiometabolic diseases is still poorly understood [1]. Irisin has been described as a protein released from skeletal muscle after physical activity [2] which may, in rodents and more controversially discussed in humans [1], mediate browning of white adipose tissue. Although the existence of circulating human irisin has even been questioned because human FNDC5 has a noncanonical ATA translation start, irisin has been detected in human plasma using mass spectrometry with appropriate control peptides [3]. IL-15 increases upon both aerobic and resistance exercise and exerts beneficial metabolic effects in patients with obesity and T2D [1]. IL-15 inhibits lipogenesis, induces fat oxidation, enhances energy expenditure, and improves insulin sensitivity and glucose metabolism [4]. To better understand the potential role of these myokines in T2D, we tested the hypotheses that IL-15 and irisin serum concentrations correlate with cardiometabolic risk parameters and are different in subgroups of T2D patients with or without diabetes complications independently of ethnicity.

## Methods

### Study design and participants

#### Korea

We included cross-sectional data from 400 Korean participants of the ongoing Korean Sarcopenic Obesity Study (KSOS) described in detail elsewhere [5]. Blood and random spot urine samples were collected in the morning after a 12 h fasting. Kidney function was assessed by estimating the eGFR using the Chronic Kidney Disease Epidemiology Collaboration formula. The urinary albumin and creatinine levels were used to calculate the urine albumin/creatinine ratio (ACR, μg/mg), and albuminuria was defined as ≥ 30 μg/mg urinary ACR. Diabetic retinopathy was diagnosed by specialized ophthalmologists from Korea University. The Korea University Institutional Review Board approved the study protocol.

#### Germany

We included a total of 400 participants (200 women, 200 men) with an established diagnosis of T2D which have been consecutively recruited at the Department of Medicine of the University Hospital in Leipzig (Germany). Patients were excluded from the analyses according to the criteria defined for the Korean subcohort. The study has been approved by the ethics committee of the University of Leipzig (approval no. 017-12-23012012), and all subjects gave written informed consent before taking part in the study. All blood samples were collected between 8 and 10 a.m. after 12 h overnight fast. T2D complications were defined as described in the Korean cohort.

### IL-15 and irisin measurements

Serum samples were analyzed centrally at Woongbee Meditech Inc., Korea. Irisin (Biovendor, Brno, Czech Republic) and IL-15 (R&D Systems Inc., Minneapolis, MN, USA) concentrations were measured using specific enzyme-linked immunosorbent assays (ELISA).

### Statistical analyses

Korean and German study populations were compared using the Mann–Whitney* U* test or independent *t* test. Fisher’s exact test or Pearson’s Chi-square was utilized to evaluate the differences in the categorical variable distribution. To investigate correlations between myokines and cardiometabolic variables, Spearman partial correlation analysis was used after adjusting for age and sex. Data were analyzed using SAS 9.2 (SAS Institute, Cary, NC, USA). A *p* value < 0.05 indicates statistical significance.

## Results

For almost all cardiometabolic risk parameters and the occurrence of T2D complications, we found significant differences between the German and Korean subcohorts (Table 1). Despite these differences, irisin and IL-15 serum concentrations were indistinguishable between German and Korean study participants, except for higher circulating irisin levels in Korean women compared to German women (*p* = 0.002). Independent of ethnicity, irisin and IL-15 serum concentrations were not correlated with most cardiometabolic risk factors (Table 2). In German participants, fat-free mass was negatively associated with irisin levels, whereas all other anthropometric and laboratory variables were not significantly correlated with irisin or IL-15 concentrations (Table 2). In Korean patients, irisin levels were negatively related to ACR levels and IL-15 concentrations only were negatively correlated with liver aminotransferases (Table 2).

**Table 1:** Interleukin-15 and irisin serum concentrations in two independent cohorts from Germany and Korea

	Men		*p*	Women		*p*
	Germans (*n* = 200)	Koreans (*n* = 213)		Germans (*n* = 200)	Koreans (*n* = 187)	
Age (years)	55.7 (51.0, 61.5)	58.0 (51.0, 66.0)	0.014	55.0 (49.1, 61.3)	60.0 (53.0, 66.0)	< 0.001
BMI (kg/m^2^)	43.0 (38.4, 49.5)	24.5 (22.8, 26.8)	< 0.001	42.4 (37.4, 50.5)	25.0 (23.0, 27.2)	< 0.001
SBP (mmHg)	134 (127, 145)	126 (118, 134)	< 0.001	134 (126, 146)	127 (115, 138)	< 0.001
DBP (mmHg)	80 (75, 86)	82 (74, 88)	0.797	82 (75, 89)	78 (72, 86)	0.001
AST (IU/L)	31.7 (25.7, 38.9)	15.0 (12.0, 22.0)	< 0.001	27.2 (22.8, 34.7)	13.0 (9.0, 20.0)	< 0.001
ALT (IU/L)	32.3 (23.4, 46.7)	18.0 (15.0, 24.0)	< 0.001	26.3 (20.4, 39.5)	18.0 (14.0, 22.0)	< 0.001
Total cholesterol (mmol/L)	4.8 (4.1, 5.5)	3.3 (2.7, 4.1)	< 0.001	5.1 (4.5, 6.0)	3.5 (2.8, 4.1)	< 0.001
HDL cholesterol (mmol/L)	1.1 (1.0, 1.3)	1.0 (0.8, 1.1)	< 0.001	1.3 (1.1, 1.5)	1.0 (0.8, 1.3)	< 0.001
Triglycerides (mmol/L)	1.8 (1.4, 2.7)	2.9 (1.9, 4.4)	< 0.001	1.7 (1.3, 2.5)	2.7 (1.9, 4.0)	< 0.001
LDL cholesterol (mmol/L)	2.8 (2.3, 3.4)	1.6 (1.2, 2.2)	< 0.001	3.1 (2.6, 3.9)	1.8 (1.3, 2.3)	< 0.001
FPG (mmol/L)	7.3 (5.9, 9.1)	5.9 (4.9, 6.9)	< 0.001	6.7 (5.3, 8.7)	5.8 (4.9, 7.1)	< 0.001
Creatinine (mg/dL)	1.1 (0.9, 1.3)	0.8 (0.6, 0.9)	< 0.001	0.9 (0.8, 1.1)	0.6 (0.5, 0.7)	< 0.001
eGFR (ml/min/1.73 m^2^)	71.2 (57, 87)	98.7 (81.5, 129.6)	< 0.001	64.5 (52.6, 76.2)	101.2 (82.7, 137.7)	< 0.001
HbA1c (%)	6.5 (5.8, 7.3)	7.1 (6.5, 7.6)	< 0.001	6.0 (5.5, 6.9)	7.1 (6.6, 7.9)	< 0.001
Smoking (*n*, %)	28 (14)	74 (34.7)	< 0.001	30 (15)	11 (5.9)	0.004
Retinopathy (*n*, %)	86 (43.0)	53 (27.7)	0.002	104 (52.0)	45 (27.1)	< 0.001
No retinopathy	114 (57.0)	138 (72.3)		96 (48.0)	121 (72.9)	
NPDR	77 (38.5)	45 (23.6)		94 (47.0)	35 (21.1)	
PDR	9 (4.5)	8 (4.2)		10 (5.0)	10 (6.0)	
Albuminuria (*n*, %)	31 (15.5)	54 (25.8)	0.010	38 (19.0)	46 (25.0)	0.155
< 30 mg/g	169 (84.5)	155 (74.2)		162 (81.0)	138 (75.0)	
30–299 mg/g	29 (14.5)	42 (20.1)		33 (16.5)	36 (19.6)	
≥ 300 mg/g	2 (1.0)	12 (5.7)		5 (2.5)	10 (5.4)	
ACR (mg/g)	4.4 (0, 17.6)	8.2 (4.8, 31.2)	< 0.001	1.3 (0., 15.8)	10.2 (5.1, 30.0)	< 0.001
Body fat (%)	36.0 (31.3, 39.5)	21.7 (19.0, 24.9)	< 0.001	46.4 (43.2, 49.8)	31.1 (27.2, 34.8)	< 0.001
Fat mass (kg)	49.2 (38.5, 60.4)	15.1 (12.3, 19.1)	< 0.001	53.0 (44.4, 64.6)	18.8 (15.4, 22.8)	< 0.001
Fat-free mass (kg)	88.8 (80.4, 98.3)	53.2 (49.8, 57.5)	< 0.001	62.0 (56.9, 67.9)	40.8 (37.5, 44.6)	< 0.001
CIMT (mm)	0.68 (0.44, 0.93)	0.78 (0.69, 0.92)	< 0.001	0.68 (0.46, 0.91)	0.72 (0.64, 0.87)	0.010
Irisin (μg/mL)	4.4 (3.5, 5.5)	4.2 (3.4, 5.7)	0.659	4.3 (3.5, 5.3)	4.6 (3.5, 7.3)	0.002
IL-15 (pg/mL)	1.1 (0.9, 1.4)	1.1 (0.8, 1.4)	0.330	1.1 (0.8, 1.3)	1.0 (0.8, 1.3)	0.762

BMI, body mass index; SBP, systolic blood pressure; DBP, diastolic blood pressure; AST, aspartate aminotransferase; ALT, alanine aminotransferase; HDL, high-density lipoprotein; LDL, low-density lipoprotein; FPG, fasting plasma glucose; eGFR, estimated glomerular filtration rate; PDR, proliferative retinopathy; NDPR, nonproliferative retinopathy; ACR, urine albumin-to-creatinine ratio; CIMT, carotid intima-media thickness; IL-15, interleukin-15

**Table 2:** Age- and sex-adjusted partial correlation analyses of irisin and IL-15 serum concentrations, clinical and laboratory parameters

	Germans				Koreans			
	Irisin		IL-15		Irisin		IL-15	
	*r*	*p*	*r*	*p*	*r*	*p*	*r*	*p*
BMI	− 0.044	0.389	− 0.050	0.326	− 0.033	0.513	0.064	0.207
SBP	0.022	0.665	0.042	0.404	0.023	0.646	0.038	0.446
DBP	0.026	0.611	0.027	0.592	0.049	0.326	− 0.022	0.668
AST	0.031	0.535	0.068	0.177	− 0.059	0.243	− 0.151	**0.003**^**a**^
ALT	0.020	0.686	0.017	0.741	− 0.033	0.517	− 0.120	**0.017**^**a**^
Total cholesterol	0.004	0.937	0.008	0.867	0.057	0.258	− 0.080	0.114
HDL cholesterol	− 0.008	0.881	0.047	0.353	0.082	0.102	− 0.061	0.230
Triglycerides	0.049	0.333	0.001	0.988	0.019	0.702	0.003	0.953
LDL cholesterol	− 0.003	0.957	− 0.017	0.731	0.038	0.454	− 0.061	0.227
FPG	− 0.015	0.765	− 0.009	0.864	0.080	0.113	− 0.022	0.667
HbA1c	− 0.064	0.204	− 0.043	0.394	− 0.026	0.603	0.018	0.716
Creatinine	− 0.043	0.394	0.001	0.990	− 0.087	0.082	0.027	0.594
eGFR	0.047	0.347	0.007	0.883	0.092	0.068	− 0.024	0.639
ACR	− 0.095	0.059	− 0.030	0.548	− 0.162	**0.001**^**a**^	0.089	0.079
Body fat	− 0.038	0.519	− 0.054	0.360	0.035	0.487	0.043	0.400
Fat mass	− 0.076	0.192	− 0.076	0.193	0.018	0.722	0.090	0.076
Fat-free mass	− 0.116	**0.047**^**a**^	− 0.084	0.151	− 0.042	0.404	0.094	0.062
CIMT	− 0.005	0.943	− 0.047	0.506	− 0.089	0.082	− 0.005	0.915

^a^After adjusting correlation coefficients to correct for multiple comparisons by Bonferroni’s correction, none of the significant correlations (bold) remained significant

BMI, body mass index; SBP, systolic blood pressure; DBP, diastolic blood pressure; AST, aspartate aminotransferase; ALT, alanine aminotransferase; HDL, high-density lipoprotein; LDL, low-density lipoprotein; FPG, fasting plasma glucose; eGFR, estimated glomerular filtration rate; ACR, urine albumin-to-creatinine ratio; CIMT, carotid intima-media thickness

In analyses of the entire study population and in the German and Korean subgroups, irisin and IL-15 serum concentrations were not significantly different between T2D patients with or without evidence for diabetic retinopathy (data not shown). Comparisons of participants with or without albuminuria only revealed significantly lower irisin (but not IL-15) serum concentrations (*p* = 0.029) in Korean T2D patients with albuminuria.

## Discussion

The key result of our study is that despite significant differences in almost all anthropometric parameters and cardiometabolic risk factors between German and Korean T2D patients, both irisin and IL-15 serum concentrations were indistinguishable. Noteworthily, Korean women displayed slightly higher irisin levels than German women. In addition, we could reproducibly show for the German and Korean cohort that irisin and IL-15 serum concentrations are not related to cardiometabolic risk factors or the presence of diabetes complications. There was only one exception that Korean T2D patients with albuminuria showed lower irisin levels compared to those without albuminuria, generating the hypothesis that there are ethnic differences in the interplay between irisin secretion or clearance and renal function. An important limitation of our study is that we only analyzed the circulating myokines at one time point. We can, therefore, not exclude that these myokines may be relevant biomarkers for the prognosis of cardiometabolic diseases and/or for the evaluation of treatment response. Another limitation is that our study combined two separate data collections into one evaluation, which may have created a potential bias.

In conclusion, our data suggest that neither irisin nor IL-15 serum concentrations reflect cardiometabolic risk factors or T2D complications in two independent cohorts from Korea and Germany.

## References

[1] Eckel J (2019). Myokines in metabolic homeostasis and diabetes. Diabetologia.

[2] Boström P, Wu J, Jedrychowski MP (2012). A PGC1-α-dependent myokine that drives brown-fat-like development of white fat and thermogenesis. Nature.

[3] Jedrychowski MP, Wrann CD, Paulo JA (2015). Detection and quantitation of circulating human Irisin by tandem mass spectrometry. Cell Metab.

[4] Ye J (2015). Beneficial metabolic activities of inflammatory cytokine interleukin 15 in obesity and type 2 diabetes. Front Med.

[5] Kim TN, Park MS, Yang SJ (2010). Prevalence and determinant factors of sarcopenia in patients with type 2 diabetes: the Korean Sarcopenic Obesity Study (KSOS). Diabetes Care.

